# Association between *GDF5* rs143383 polymorphism and knee osteoarthritis: an updated meta-analysis based on 23,995 subjects

**DOI:** 10.1186/1471-2474-15-404

**Published:** 2014-12-02

**Authors:** Feng Pan, Jing Tian, Tania Winzenberg, Changhai Ding, Graeme Jones

**Affiliations:** Menzies Research Institute Tasmania, University of Tasmania, Private Bag 23, Hobart, Tasmania 7000 Australia

**Keywords:** GDF5, Polymorphism, Knee, Osteoarthritis, Meta-analysis

## Abstract

**Background:**

Previous studies investigating the association between GDF5 rs143383 polymorphism and knee osteoarthritis (OA) have suggested stronger associations in Asians than Caucasians, but limitations on the amount of available data have meant that a definitive assessment has not been possible. Given the availability of more recent data, the aim of this meta-analysis was to determine the overall association between GDF5 rs143383 polymorphism and knee OA and whether the association varies by ethnicity.

**Methods:**

Searches of Medline, Embase, and ISI Web of Science were conducted up to July 2013. Summary odds ratios (ORs) and 95% confidence intervals (CIs) were calculated to estimate the strength of association between the GDF5 polymorphism and knee OA risk.

**Results:**

A total of 20 studies with 23,995 individuals were included. There were weak but significant associations present between the GDF5 polymorphism and knee OA at the allele level (C vs. T: OR =0.85, 95% CI = 0.80-0.90) and genotype level (CC vs. TT: OR = 0.73; CT vs. TT: OR = 0.84; CC/CT vs. TT: OR = 0.81; CC vs. CT/TT: OR = 0.81) in the overall population. In the subgroup analysis by ethnicity, we observed a strong significant association (OR = 0.60 to 0.80, all *P* <0.05) in Asian population and weaker associations (OR =0.78 to 0.87, all *P* <0.05) in Caucasian population; however marked heterogeneity was detected in all models except for CC vs. TT (*I*^*2*^ = 12.9%) and CC vs. CT + TT (*I*^*2*^ = 0.0%) in Asians.

**Conclusions:**

These results strongly suggest that the C allele and CC genotype of the GDF5 gene are protective for knee OA susceptibility across different populations.

**Electronic supplementary material:**

The online version of this article (doi:10.1186/1471-2474-15-404) contains supplementary material, which is available to authorized users.

## Background

Osteoarthritis (OA) is the most prevalent form of arthritis in the worldwide and is regarded as a disorder of the whole joint [1, 2]. One of the most frequently affected joints is the knee, with a prevalence of 30% in those over 65 years old [3]. There is a strong genetic component of OA with heritability estimates showing that genetic components account for 39-65% of the risk for the development of knee OA [2, 4, 5]. However, the identification of specific genes has been problematic with some genes associated with pain [6, 7] and others with joint structures [8]. Overall, there is a lack of consistency of associations.

One of the most comprehensively studied candidate genes is growth differentiation factor 5 (GDF5). GDF5, also known as cartilage-derived morphogenetic protein 1, is a member of the transforming growth factor-β superfamily and closely correlated with bone morphogenetic proteins. GDF5 has been shown to be involved in musculoskeletal processes including the development, maintenance and repair of bone, cartilage and other tissues of synovial joint as well as tendon [9, 10]. In light of the important functions of GDF5, any changes affecting a reduction in the expression of this protein could increase the risk of OA.

GDF5 mutations in humans have been implicated in several disorders of skeletal development [11]. Single-nucleotide polymorphisms (SNPs) have been identified in the 5′-untranslated region (5′-UTR) of GDF5 which is involved in the regulation of GDF5 transcriptional activity [12]. As for one of the most common polymorphisms (rs143383), T to C substitution in the promoter region of GDF5 has an effect on the expression of GDF5 production, with lower GDF5 expression of the OA-associated T allele [13]. Several studies have suggested that GDF5 rs143383 polymorphism may be related to an elevated risk of OA in certain ethnic groups [12, 14–16]. However, these positive associations have not been consistently replicated. For instance, two studies from Korea and Greece failed to detect any association with knee OA [17, 18]. Two earlier meta-analyses suggested that an increase in the risk of knee OA was associated with GDF5 rs143383 polymorphism in Asians and Caucasians [11, 19]. Since then, multiple studies on the relationship of knee OA with GDF5 have been published. Therefore, the aim of this study was to determine the overall association between GDF5 rs143383 polymorphism and knee OA risk and whether the association varies by ethnicity.

## Methods

### Literature search strategy

We searched Medline, Embase and ISI Web of science databases for all English articles on the association between GDF5 gene promoter polymorphism and OA (last report up to 13 July 2013). Combinations of keywords used in the search were: (“Growth differentiation factor 5” or “GDF5” or “rs143383” or “+104 T/C”), (“polymorphism” or “polymorphisms”) and (“osteoarthritis” or “OA”). References of retrieved studies and review articles were also screened for other additional eligible publications and unpublished studies. Conference abstracts were not considered.

### Inclusion and exclusion criteria

All studies included in this meta-analysis had to meet the following criteria: (1) the type of study was a case–control or cohort study; (2) a study investigated the association of GDF5 (rs143383; +104 T/C) polymorphism with knee OA; (3) available alleles or genotypes frequencies of GDF5 were provided to evaluate the odds ratios (ORs) with 95% confidence intervals (CIs). The exclusion criteria were as follows: (1) the study was not conducted on knee OA; (2) the study was conducted on animals or cells; (3) the data could not be extracted after contacting with the authors.

### Data extraction

All data were extracted independently from eligible studies by two reviewers (Pan and Tian) according to the criteria listed above. The following information were collected: the first author’s name, publication date, country of origin, study design, ethnicity, total sample size of cases and controls, genotype and allele frequencies of cases and controls, sources of controls, age, sex and genotyping method, which also were reviewed by a third investigator (Jones). We also extracted data on how knee OA was defined i.e. clinical criteria, radiographic criteria, or total knee replacement (TKR). For clinical criteria, the American College of Rheumatology (ACR) criteria was used if information on ACR was available [20]. For radiographic criteria, Kellgren/Lawrence (K/L) score (0–4 scale) was used to identify and grade knee OA. A cut-off of K/L score 2 was used to be a classification of knee OA [21]. Any controversies about interpretation of data were discussed within our research team to reach a consensus. In cases where the same patient population was included in different studies, only the larger sample size was included in this meta-analysis. If one study contains the results from different populations, each group was treated independently. Authors were contacted where unpublished data or clarification were needed.

### Statistical methods

Allele frequencies at GDF5 rs143383 polymorphism from the respective study were determined by the allele counting method. The strength of the association between GDF5 rs143383 polymorphism and knee OA susceptibility was estimated by calculating the pooled ORs with their 95% CIs. The Z-test was used to determine the significance of the pooled ORs and 95% CIs. The pooled ORs were performed for additive (C vs. T), co-dominant (CC vs. TT; CT vs. TT), dominant (CC + CT vs. TT), and recessive (CC vs. CT + TT) models. The between-study heterogeneity was assessed using the Chi square based Q-statistic [22]. If a *P* value less than 0.10 for the *Q*-test was observed, it indicates the presence of heterogeneity among studies [23]. The *I*^*2*^ statistic (*I*^*2*^ = 100% × (*Q*-df)/*Q*) was also used to quantify heterogeneity. *I*^*2*^ ranges from 0 to 100% which is interpreted as the degree of inconsistency across studies [24]. An *I*^*2*^ greater than 50% was considered as heterogeneity among studies. The random-effect model was used to determine the pooled ORs. Sensitivity analysis was performed by excluding the Hardy-Weinberg equilibrium (HWE)-violating studies [25]. Potential publication bias was assessed by the funnel plot, in which the standard error of log (OR) of each study was plotted against its log (OR). A symmetric plot indicates a low risk of publication bias. If visual inspection suggested there was funnel plot asymmetry, the method of Egger’s linear regression test was used to further assess [26]. All statistical analyses were carried out using STATA version 7.0 (Stata Corporation, College Station, TX). Two-sided *P* <0.05 was considered to be statistically significant.

## Results

### Characteristics of included studies

**Figure 1:**
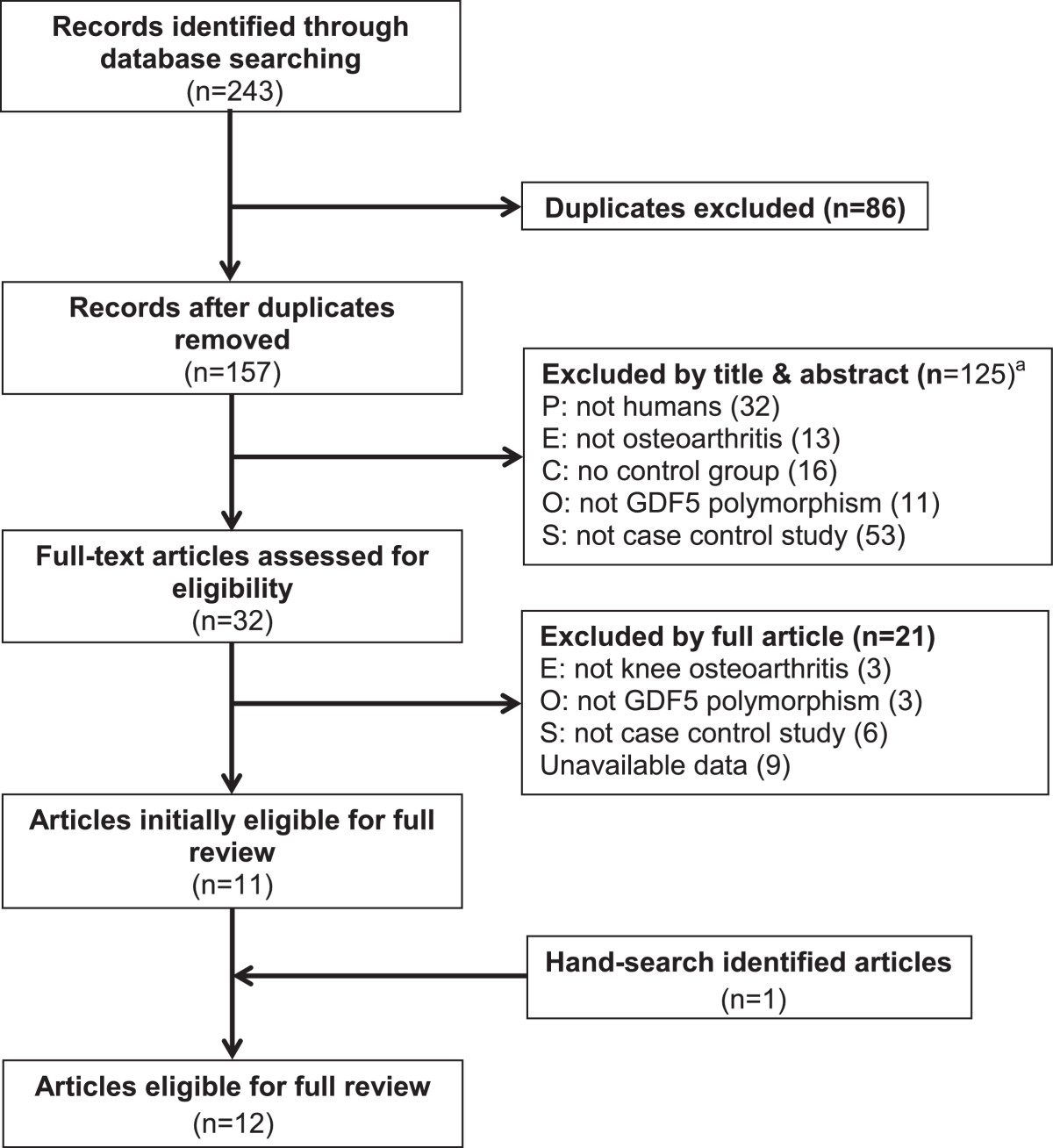
**Flow diagram of the study selection process.** ^a^P: population group; E: exposure; C: control group; O: outcome; S: study design.

A total of 12 articles were identified [11, 12, 14–19, 27–30]. Among these, one article [12] reported on a Japanese population and a Chinese population, these were considered as two separate studies. Two other studies performed by Southam et al. [14] and Valdes et al. [27] also included two independent studies, the former contained UK and Spanish studies, and the latter investigated two different populations in the UK. In addition, the three previous meta-analyses included unpublished data from independent studies [11, 19, 30] where only T allele and C allele counts can be extracted from the Twins UK and Finnish study [19], and the Rotterdam study III [30]. Thus, 20 studies with 8,709 cases and 15,286 controls were included in the current meta-analysis. The study flow chart is shown in Figure 1. Detailed characteristics of these studies are listed in Tables 1 and 2. Of eligible studies, 6 studies (n = 6,219) and 14 studies (n = 17,776) were conducted respectively in Asian and Caucasian populations. Male and female subgroups were available from 5 studies in Asian and 4 studies in Caucasian population.

**Table 1:** **Characteristics of individual studies included in meta-analysis**

First author	Year	Country	Study design	Ethnicity	Sample size		Source of controls	Genotyping method	Age (mean)		Knee OA definition		
					Case	Control			Case	Control	Radiographic*	Clinical^†^	TKR
Southam^a^	2007	UK	Case–control	Caucasian	349	822	HB	PCR-RFLP	65	69			**+**
Southam^b^	2007	Spain	Case–control	Caucasian	274	1196	HB	TaqMan	NA	>55.0			**+**
Miyamoto^a^	2007	Japan	Case–control	Asian	718	861	HB	TaqMan, Invader, DNA fragment analysis or Direct sequence	71.9	49.4	**+**		
Miyamoto^b^	2007	China	Case–control	Asian	313	485	HB	TaqMan, Invader, DNA fragment analysis or Direct sequence	58.8	56.8	**+**		
Tsezou	2007	Greece	Case–control	Caucasian	251	268	HB	Direct sequence	67.9	65.2	**+**		
Chapman	2008	Netherlands	Cohort study	Caucasian	142	724	PB	Mass spectrometry	60.4	59.6	**+**		
Valdes^a^	2009	UK	Case–control	Caucasian	735	654	HB	Allele-specific PCR	68.5	66.9		**+**	
Valdes^b^	2009	UK	Cohort study	Caucasian	264	512	PB	Allele-specific PCR	66.3	63	**+**		
Vaes	2009	Netherlands	Cohort study	Caucasian	667	2097	PB	TaqMan	>55.0	>55.0	**+**		
Evangelou^a^	2009	Iceland	Cohort study	Caucasian	1071	1169	PB	Centaurus platform	74.8	74.8			**+**
Evangelou^b^	2009	UK	Twins study	Caucasian	177	548	NA	Illumina platform	54.3	54.3	**+**		
Evangelou^c^	2009	Finland	Family-based study	Caucasian	109	209	NA	Mass spectrometry	67	58	**+**		**+**
Cao	2010	Korea	Case–control	Asian	276	298	PB	PCR-RFLP	63	44			**+**
Tawonsawatruk	2011	Thailand	Case–control	Asian	103	103	HB	PCR-RFLP	68.5	59.25			**+**
Valde^a^	2011	UK	Cohort study	Caucasian	867	758	PB	Allele-specific PCR	66.5	66.5			**+**
Valdes^b^	2011	Estonian	Cohort study	Caucasian	65	427	PB	Allele-specific PCR	47.1	47.1	**+**		
Valdes^c^	2011	UK	Cohort study	Caucasian	1141	536	PB	Allele-specific PCR	65.5	65.5	**+**		**+**
Valdes^d^	2011	Netherlands	Cohort study	Caucasian	162	1582	PB	TaqMan	>45.0	>45.0	**+**		
Shin	2012	Korea	Cohort study	Asian	725	1737	PB	High resolution melting analysis	67.4	62.7	**+**		
Mhishra	2013	India	Case–control	Asian	300	300	HB	PCR-RFLP	54.0	55.2	**+**	**+**	

^a,b,c^ and ^d^Denote an independent study in the one article, respectively; NA Data not available; HB hospital-based; PB population-based; PCR-RFLP polymerase chain reaction restriction fragment length polymorphism; TKR total knee replacement.

*Radiographic criteria (Kellgren/Lawrence grade ≥2).

^†^Clinical criteria are based on the American College of Rheumatology.

**Table 2:** **Distributions of GDF5 rs143383 genotypes and alleles among cases and controls**

First author	Year	Case			Control			Case		Control		***P*** ^***HWE***^
		TT	TC	CC	TT	TC	CC	T	C	T	C	
Southam^a^	2007	141	168	40	324	372	126	450	248	1020	624	0.262
Southam^b^	2007	102	136	36	439	563	194	340	208	1441	951	0.550
Miyamoto^a^	2007	444	243	31	473	330	58	1131	305	1276	446	0.966
Miyamoto^b^	2007	197	97	19	244	193	48	491	135	681	289	0.283
Tsezou	2007	95	126	30	99	125	44	316	186	323	213	0.669
Chapman	2008	54	72	16	289	331	104	180	104	909	539	0.558
Valdes^a^	2009	337	313	85	238	329	79	987	483	805	487	0.032
Valdes^b^	2009	126	98	35	181	244	84	350	168	606	412	0.908
Vaes	2009	276	298	93	752	1014	331	850	484	2518	1617	0.724
Evangelou^a^	2009	535	379	157	552	442	175	1449	693	1546	792	0.000
Evangelou^b^	2009	NA	NA	NA	NA	NA	NA	230	124	679	417	NA
Evangelou^c^	2009	NA	NA	NA	NA	NA	NA	124	94	251	167	NA
Cao	2010	150	115	11	159	113	26	415	137	431	165	0.360
Tawonsawatruk	2011	38	41	11	33	47	23	117	63	113	93	0.424
Valdes^a^	2011	413	361	93	294	354	110	1187	547	942	574	0.837
Valdes^b^	2011	32	24	9	168	179	80	88	42	515	339	0.010
Valdes^c^	2011	467	511	163	219	237	80	1445	837	675	397	0.229
Valdes^d^	2011	NA	NA	NA	NA	NA	NA	195	107	1930	1234	NA
Shin	2012	382	305	38	942	689	106	1069	381	2573	901	0.176
Mhishra	2013	124	130	46	84	160	56	378	222	328	272	0.188

^a,b,c^ and ^d^Denote an independent study in the one article, respectively; HWE Hardy-Weinberg equilibrium; NA Data not available.

### Quantitative assessment

The summary of meta-analyses for GDF5 rs143383 polymorphism with OA is shown in Table 3.

**Table 3:** **Meta-analysis of GDF5 rs143383 polymorphism and knee OA**

Population	Comparison (N^a^)	Test of association		Test of heterogeneity	
		OR (95% CI)	***P*** ^***b***^	***P*** ^***c***^	***I*** ^***2***^(%)
Overall	C vs. T (20)	0.85 (0.80-0.90)	0.000	0.019	44.1
	CC vs. TT (17)	0.73 (0.66-0.81)	0.000	0.328	10.8
	CT vs. TT (17)	0.84 (0.76-0.94)	0.002	0.000	62.2
	CC/CT vs. TT (17)	0.81 (0.73-0.90)	0.000	0.000	61.7
	CC vs. CT/TT (17)	0.81 (0.74-0.86)	0.000	0.623	0.0
Ethnicity					
Asian	C vs. T (6)	0.78 (0.67-0.92)	0.003	0.006	69.5
	CC vs. TT (6)	0.60 (0.48-0.76)	0.000	0.333	12.9
	CT vs. TT (6)	0.80 (0.63-1.02)	0.071	0.002	74.3
	CC/CT vs. TT (6)	0.76 (0.60-0.96)	0.021	0.001	75.8
	CC vs. CT/TT (6)	0.68 (0.56-0.84)	0.000	0.494	0.0
Caucasian	C vs. T (14)	0.87 (0.82-0.92)	0.000	0.229	20.7
	CC vs. TT (11)	0.78 (0.70-0.87)	0.000	0.611	0.0
	CT vs. TT (11)	0.86 (0.76-0.97)	0.012	0.011	56.2
	CC/CT vs. TT (11)	0.83 (0.75-0.93)	0.001	0.020	52.6
	CC vs. CT/TT (11)	0.84 (0.76-0.93)	0.001	0.822	0.0
Sex					
Females	C vs. T (9)	0.85 (0.78-0.93)	0.000	0.236	23.3
	CC vs. TT (9)	0.73 (0.62-0.87)	0.000	0.923	0.0
	CT vs. TT (9)	0.81 (0.69-0.95)	0.011	0.029	53.2
	CC/CT vs. TT (9)	0.80 (0.69-0.93)	0.003	0.039	50.7
	CC vs. CT/TT (9)	0.83 (0.70-0.97)	0.021	0.990	0.0
Males	C vs. T (9)	0.85 (0.74-0.97)	0.020	0.171	30.9
	CC vs. TT (9)	0.65 (0.50-0.84)	0.001	0.464	0.0
	CT vs. TT (9)	0.99 (0.81-1.20)	0.888	0.139	34.8
	CC/CT vs. TT (9)	0.90 (0.74-1.09)	0.272	0.107	39.1
	CC vs. CT/TT (9)	0.66 (0.51-0.84)	0.001	0.613	0.0

OR odds ratio, CI confidence interval, ^a^Number of comparison, ^b^ *P* values for within group differences were determined by Z test, ^c^ *P P* value of *Q*-test for heterogeneity test.

### Overall population

**Figure 2:**
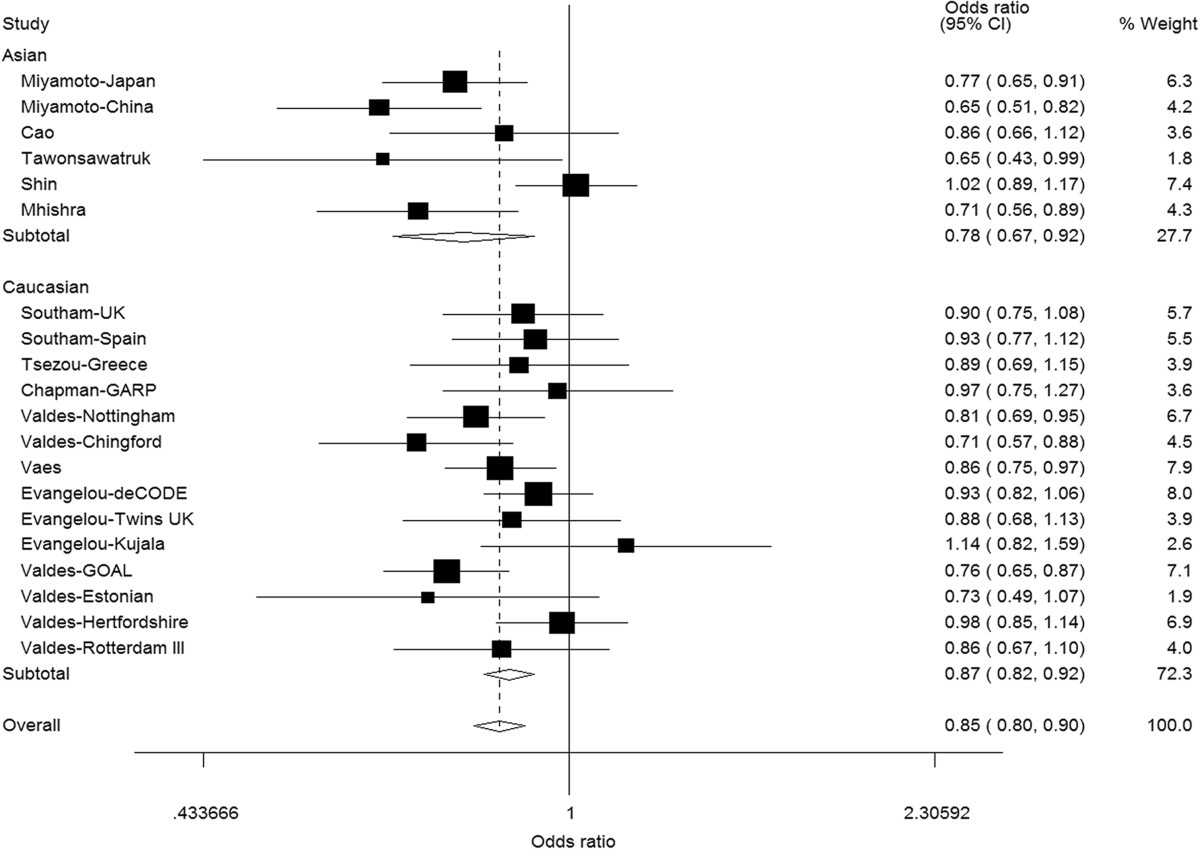
**Forest plot of the association of GDF5 rs143383 polymorphism with knee osteoarthritis risk under additive model (C versus T).**

20 separate studies had available data for analysis of GDF5 rs143383 polymorphism and knee OA risk with a total sample size of 8,709 cases and 15,286 controls. In the allele model and genotype models, significant associations were found when all studies were pooled in the overall population (Table 3). The summary OR for allele model was 0.85 (95% CI = 0.80-0.90). The forest plot of the distribution of the ORs for allele model is shown in Figure 2. Similarly, the summary ORs for genotype models ranged from 0.73 to 0.84. There was substantial and statistically significant heterogeneity for CT vs. TT (*I*^*2*^ = 62.2%) and dominant model (*I*^*2*^ = 61.7%).

### Subgroup analyses by ethnicity

**Figure 3:**
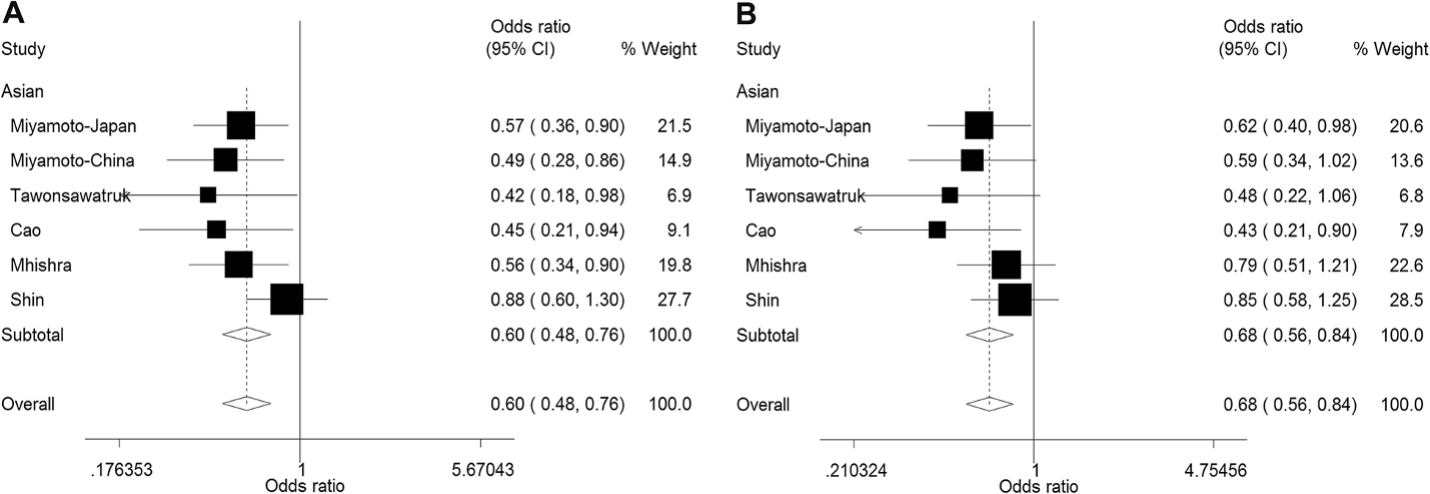
**Forest plots for statistically significant meta-analysis in Asian populations. (A)** CC versus TT; **(B)** CC versus CT/TT.

Protective effects in Asian populations were consistently greater in magnitude and the associations were all statistically significant in the Asian subgroup except for CT versus TT which approached but did not reach significance (*P* = 0.071) (Table 3). The summary ORs were highly significant especially for CC vs. TT (OR = 0.60, *P* <0.001) and the recessive model (OR = 0.68, *P* <0.001) (Figure 3). In the Asian subgroup, the between-study heterogeneity remained substantial apart from two models (CC vs. TT and the recessive model, *I*^*2*^ = 12.9% and *I*^*2*^ = 0.0%, respectively). In Caucasian populations, similar results were found under all models with weaker associations (OR = 0.78 to 0.87, all *P* <0.05), but a lower heterogeneity was observed.

### Subgroup analyses by sex

**Figure 4:**
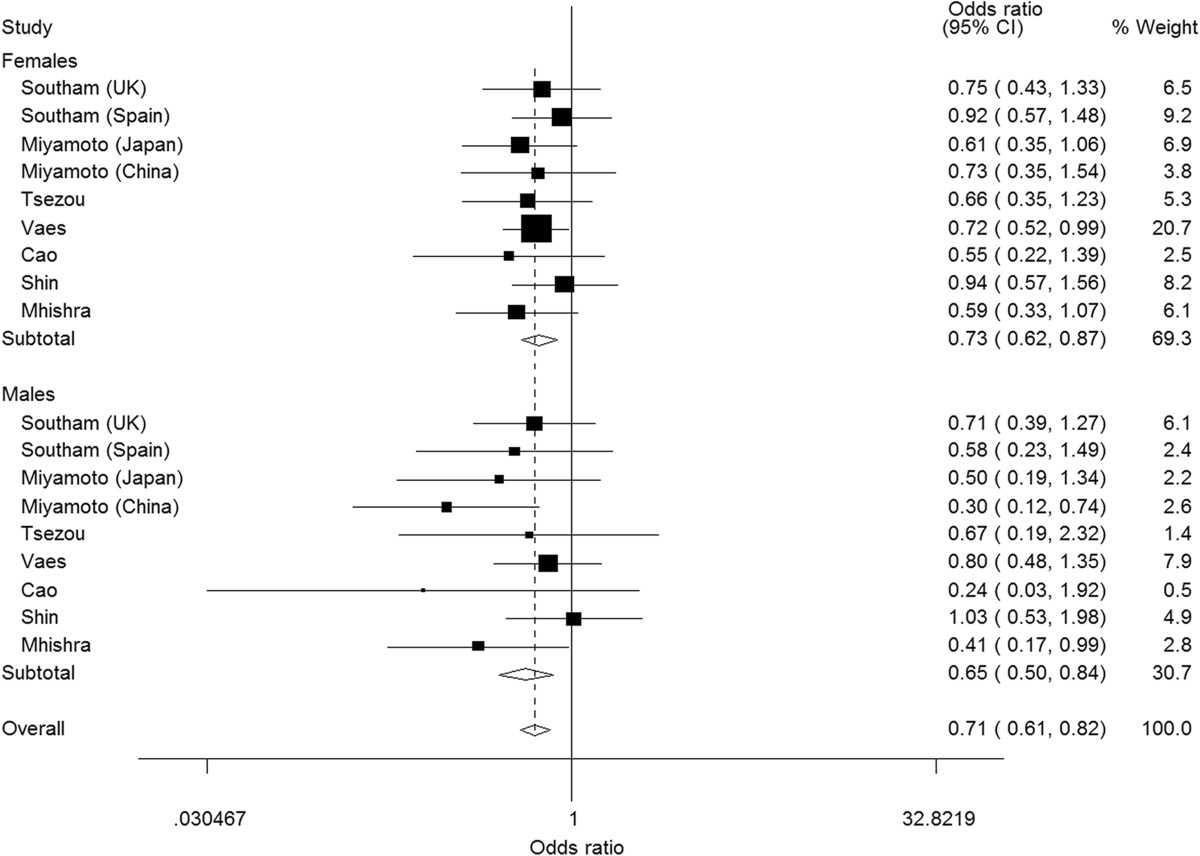
**Subgroup analysis by sex for knee osteoarthritis risk associated with GDF5 rs143383 polymorphism under CC versus TT model.**

When 9 studies with a sample size of females (n = 7,203) and males (n = 4,733) were stratified by sex, there were no significant differences in effects between males and females (Table 3). In females, all models showed significant associations. A stronger significant association was observed for CC vs. TT (OR = 0.73, *P* <0.001) in comparison with other models in males, similarly, there were significant differences for CC vs. TT and recessive model in females with the strongest association being for CC vs. TT (OR = 0.65, *P* = 0.001) (Figure 4). Furthermore, not all associations were significant in males (CT vs. TT: OR = 0.99, 95% CI = 0.81-1.20; CC/CT vs. TT: OR = 0.90, 95% CI = 0.74-1.09). Intriguingly, stratification by sex reduced heterogeneity in both males and females in all models compared to that seen in the overall population. In females, substantial and statistically significant heterogeneity persisted only for CT vs. TT (*I*^*2*^ = 53.2%) and dominant models (*I*^*2*^ = 50.7%). In males, *I*^*2*^ < 50% was observed in all models.

### Evaluation of other potential sources of heterogeneity

In addition to evaluation of sources of heterogeneity by ethnicity and sex, we also further investigated other potential sources of heterogeneity by control types and knee OA definition (Table 4). Subgroup analysis by control types found that heterogeneity of hospital-based group was partly attenuated with *I*^*2*^ = 0% for CC vs. TT and recessive model; however, significant heterogeneity still was seen in the population-based group. When stratification by knee OA definition, a significant reduction in the heterogeneity (*I*^*2*^ < 41.0%) was observed where TKR was used to define the cases but not for those studies using radiographic criteria.

**Table 4:** **Identifying the source of heterogeneity by control type and knee OA definition**

Subgroup	C vs. T		CC vs. TT		CT vs. TT		CC/CT vs. TT		CC vs. CT/TT	
	*P*_*h*_	*I* ^*2*^(%)	*P*_*h*_	*I* ^*2*^(%)	*P*_*h*_	*I* ^*2*^(%)	*P*_*h*_	*I* ^*2*^(%)	*P*_*h*_	*I* ^*2*^(%)
**Source of controls**										
HB	0.196	29.1	0.681	0.0	0.013	60.8	0.032	54.4	0.703	0.0
PB	0.051	46.6	0.233	23.7	0.006	62.9	0.005	64.0	0.511	0.0
**Knee OA definition**										
Radiographic	0.043	48.3	0.654	0.0	0.003	68.1	0.002	68.9	0.874	0.0
TKR	0.206	30.5	0.132	41.0	0.187	33.3	0.184	33.6	0.153	38.0

HB hospital-based, PB population-based, TKR total knee replacement, *P*_*h,*_ *P* value of *Q*-test for heterogeneity test.

### Sensitivity analyses

Sensitivity analyses were performed by excluding the HWE-violating studies to evaluate the stability of the results. Departure from HWE was observed in the controls of three studies (Table 2). After excluding these studies, the corresponding ORs did not materially alter under all models, suggesting that the results of this meta-analysis are stable (data not shown).

### Evaluation of publication bias

**Figure 5:**
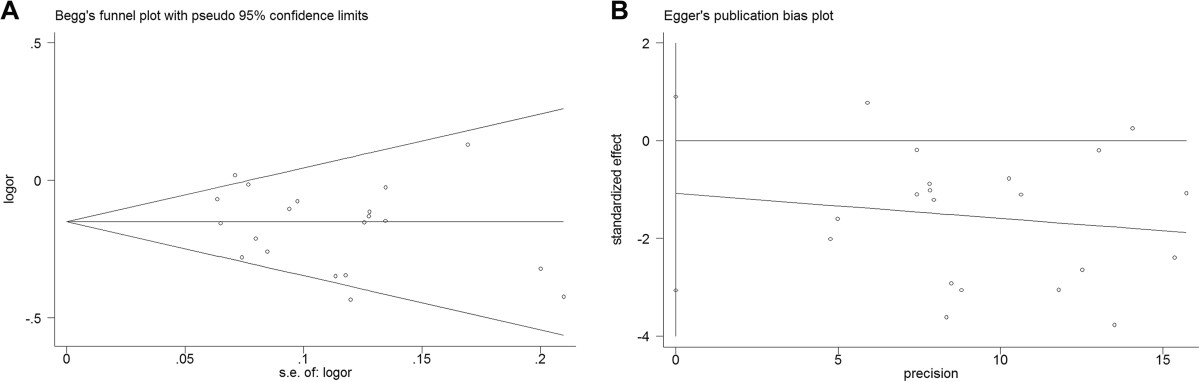
**Funnel plot and Egger’s publication bias plot for meta-analysis on association between GDF5 rs143383 polymorphism and knee osteoarthritis risk (C versus T). (A)** Begg’s funnel plot for meta-analysis; **(B)** Egger’s linear regression test for publication bias.

Begger’s funnel plot was firstly performed to assess the publication bias. As shown in Figure 5, no obvious asymmetry was found by the shape of the funnel except for CC vs TT and recessive model. Egger’s test was then performed for statistical test, revealing there might be publication bias under CC versus TT and recessive model.

## Discussion

To our knowledge, this is the largest and most comprehensive meta-analysis to assess the association of GDF5 rs143383 polymorphism with knee OA, including data from 20 studies in 8,709 knee OA cases and 15,286 controls. Overall analysis of pooled results demonstrated a statistically significant association between the variant genotype of GDF5 and knee OA risk in all comparisons. When stratification by ethnicity, significant associations were found in Asian as well as in Caucasian populations with a greater effect sizes in Asian population, suggesting that GDF5 rs143383 polymorphism is a determinant for knee OA risk and shared between Asian and Caucasian populations.

GDF5, an extracellular signalling molecule, plays a critical role in the development, maintenance and repair of synovial joint tissues, and it has been suggested that deficiency of GDF5 is one of the most important risk factors for the pathogenesis of OA [10]. The expression of the GDF5 protein is modulated by the GDF5 gene, and rare deleterious mutations in the GDF5 gene cause several disorders of skeletal development, such as chondrodysplasias and brachydactyly, suggesting this gene has a crucial role in joint homeostasis and repair [17]. Several animal models have further confirmed the evidence supporting a critical role of GDF5 [31–34]. In mice with GDF5 mutation, a number of abnormalities of joint were found including the decrease in appendicular skeleton and the limb long bones, soft tissue deformities and tendon anomaly. Taken together, these results imply that GDF5 polymorphism may have an important function in the aetiology and pathogenesis of OA.

In this study, we found that C allele of GDF5 was protective for knee OA susceptibility (OR = 0.85, 95% CI = 0.80-0.90, *P* <0.001), and T allele of GDF5 was associated with a higher risk for knee OA development. These findings seem to be biologically plausible. The T allele of the rs143383 SNP has been shown to be associated with a reduction in GDF5 transcriptional activity, thereby increasing the risk of developing knee OA, compared with the GDF5 C allele [12, 14]. In the subgroup analysis by ethnicity, effects sizes were consistently greater in Asian populations as compared to Caucasian populations, indicating that the same gene polymorphism may have different roles in knee OA susceptibility among different racial backgrounds, and the difference in linkage disequilibrium patterns may exist [19].

Several meta-analyses have been performed to identify the association between the GDF5 variant and knee OA risk. In a previous meta-analysis by Chapman et al. [11] including 2,207 cases and 4,356 controls, a significant association of GDF5 rs143383 polymorphism with knee OA was observed in Asians as well as Caucasians. In another meta-analysis, Evangelou et al. [19] included 5,085 cases and 8,135 controls and found that GDF5 rs143383 polymorphism was associated with the risk of knee OA. In the subgroup analysis, the same association was observed across different populations. Although two recent meta-analyses also suggested that GDF5 rs143383 polymorphism was associated with knee OA susceptibility, their results should be interpreted with caution [35, 36]. In the study performed by Liu et al. [35], all OA cases were pooled into their study and subgroup analyses by joint site, ethnicity, and sex were not performed. In the study by Hao and Jin [36] comprising 6 studies with 2,744 cases and 4,518 controls, there was incomplete identification of publications, which may distort the results [37]. Valdes et al. [30] also performed the meta-analysis with the largest sample size (7,579 cases and 11,947 controls), reporting that the T allele of the GDF5 polymorphism is associated with a 17% elevated risk for knee OA. Consistent with this, the present meta-analysis with a larger sample size showed a similar effect size of T allele for knee OA in the overall population. However, we found a slightly lower risk estimate for the T allele (OR = 1.15) in Caucasian population as compared to the Valdes’ paper (OR = 1.16). This discrepancy may be due to inconsistency of reporting data from the Rotterdam I study. In the current paper, data from this study was extracted from one of the original papers reporting Rotterdam I study [28] rather than from a previous meta-analysis [19], thereby leading to a slight data variation. Additionally, the GDF5 polymorphism was found to be consistently associated with knee OA risk in Asian population. This further provides strong evidence of GDF5 rs143383 polymorphism to knee OA risk across different populations.

Heterogeneity is a potential problem in the understanding the results of meta-analyses. In this study, significant heterogeneity between different studies was observed in the overall population. To clarify the source of heterogeneity, ethnicity and sex were used to stratify the studies, finding part of this heterogeneity can be effectively attenuated or removed when stratification by sex. This indicates that it is important for meta-analyses of genetic association studies to perform subgroup analyses by sex. After subgroup analysis by source of controls, the heterogeneity was also decreased; therefore, it can be assumed that the heterogeneity partly results from difference of source of controls. That may be because potential confounding factors in many epidemiologic studies may result from the difference in control types [38]. In addition, different studies used different criteria to define the cases, which might be one of sources of heterogeneity. Some centres defined their cases using the K/L classification and/or ACR criteria, whereas other centres used a TKR to define their cases. These differences between studies in the control group as well as key characteristics of the participants might lead to heterogeneity in the magnitude of the genetic effects [19]. Therefore, a broad consensus should be reached about OA phenotype definitions and how to enrol an ideal control group. Furthermore, other factors also should be explored to identify the source of heterogeneity if more data was available.

Of note, several potential limitations of this study should be acknowledged. Firstly, knee OA is a multifactorial disease with complex associations between genetic factors and environmental factors, and is a polygenic disease that could not be conferred significantly by no loci individually [39, 40]. Hence, some environmental factors or other polymorphic loci should be taken into account together to arrive at a true effect of GDF5 gene. Secondly, in view of our results from unadjusted estimates, a more accurate assessment should be performed according to age, body mass index, smoking status, and other lifestyle factors if more detailed data were available. Thirdly, publication bias was found in two models, which may give rise to biased results, in particular potentially an overestimate of the effect. However, unpublished studies would need to have a large negative association to have sufficient weight to substantially change our results.

## Conclusions

This meta-analysis suggests that GDF5 rs143383 polymorphism is highly associated with the susceptibility to knee OA with protective associations for the C allele and CC genotype across different populations.

## Authors’ original submitted files for images

Below are the links to the authors’ original submitted files for images. Authors’ original file for figure 1 Authors’ original file for figure 2 Authors’ original file for figure 3 Authors’ original file for figure 4 Authors’ original file for figure 5
