# Supplementary figures and images for: Association between GDF5 rs143383 polymorphism and knee osteoarthritis: an updated meta-analysis based on 23,995 subjects
Source: BMC Musculoskelet Disord. 2014 Dec 2;15:404. doi: 10.1186/1471-2474-15-404 (PMC4265459; doi:10.1186/1471-2474-15-404)

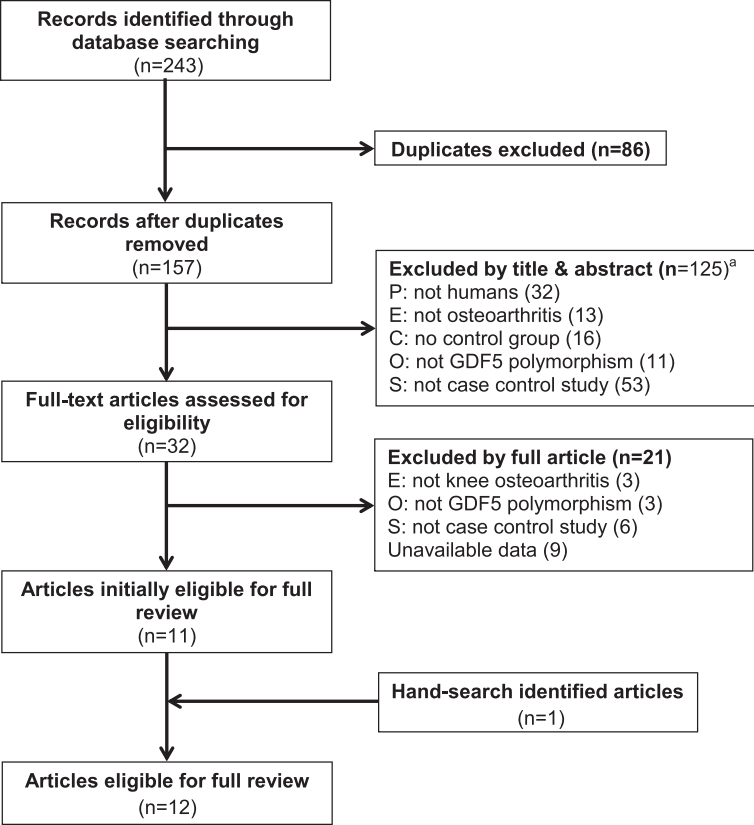

Supplement: Supplementary file 1 — Authors’ original file for figure 1 [file 12891_2013_2342_MOESM1_ESM.pdf]

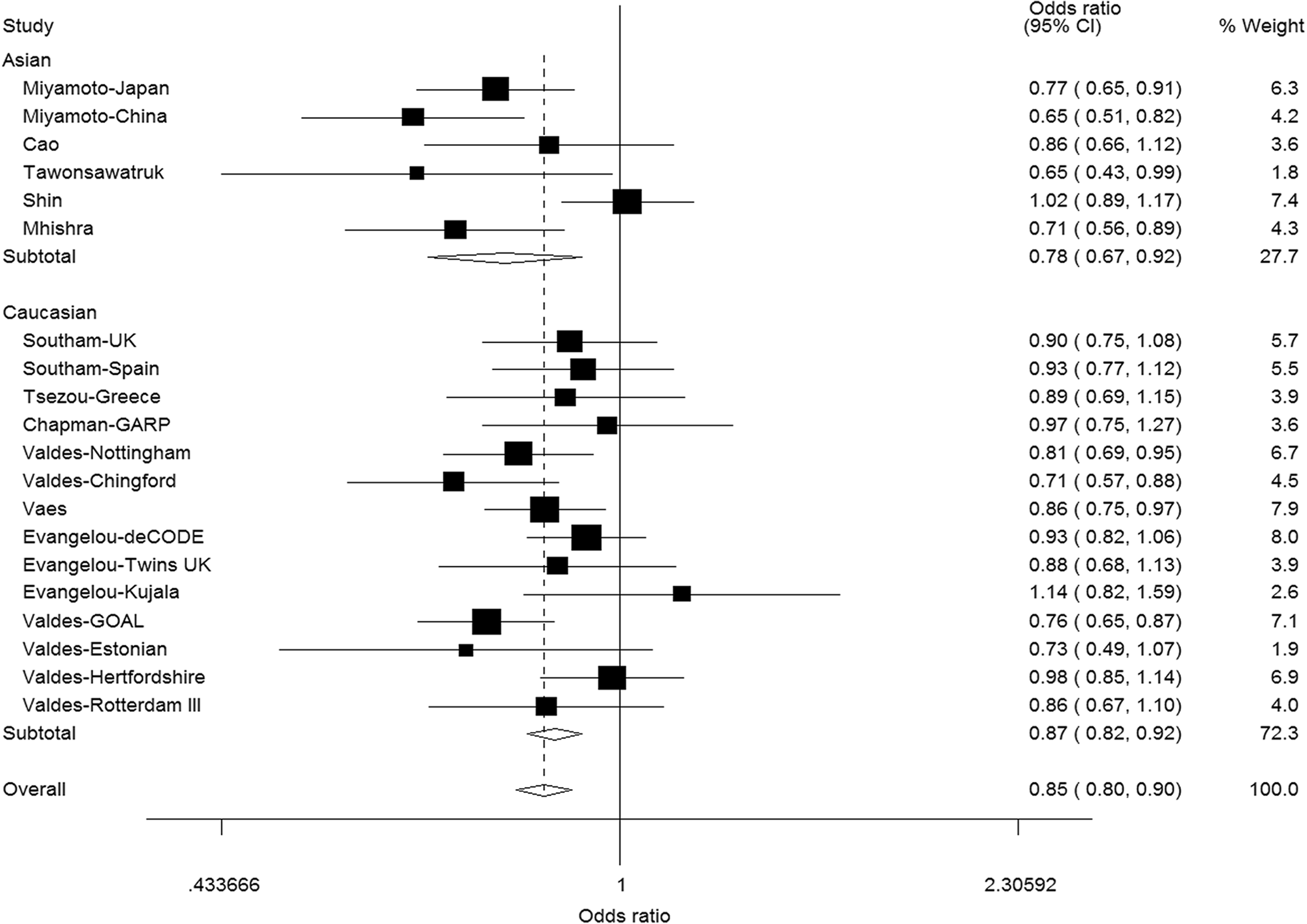

Supplement: Supplementary file 2 — Authors’ original file for figure 2 [file 12891_2013_2342_MOESM2_ESM.tif]

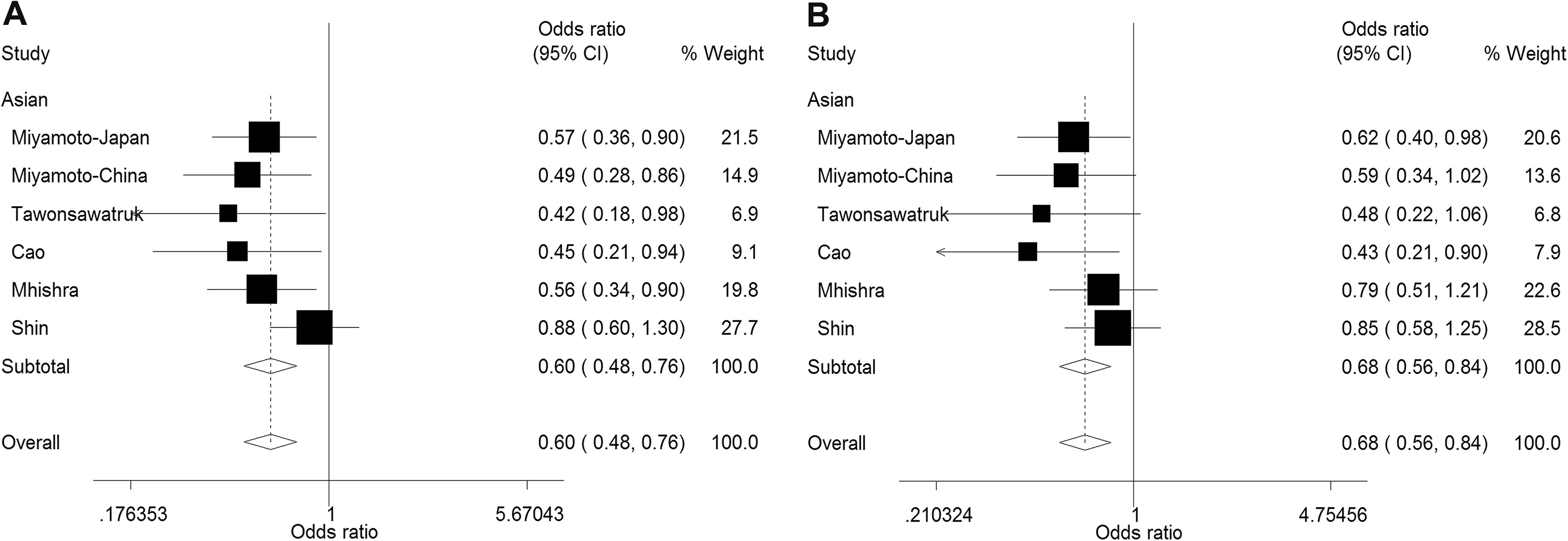

Supplement: Supplementary file 3 — Authors’ original file for figure 3 [file 12891_2013_2342_MOESM3_ESM.tif]

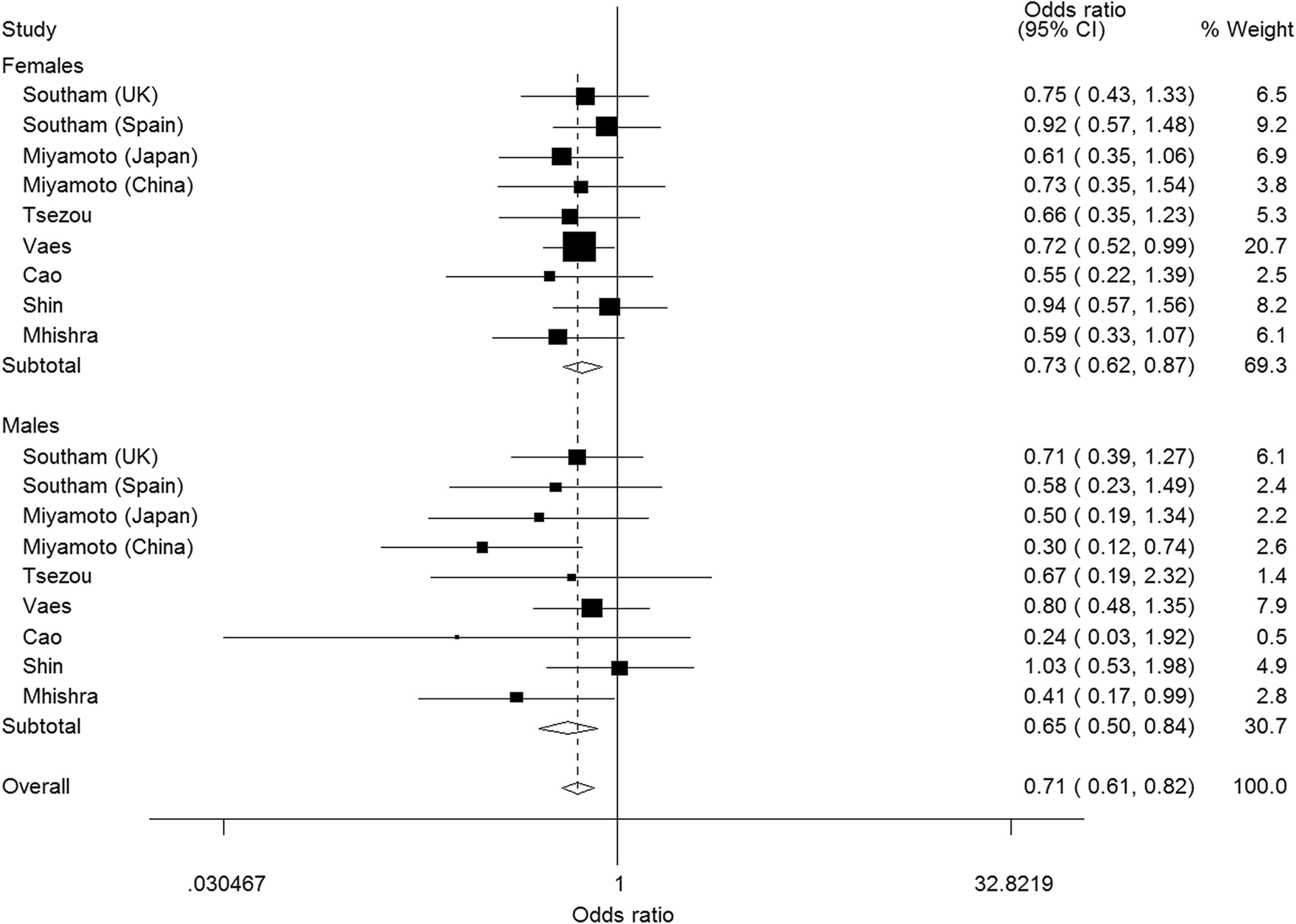

Supplement: Supplementary file 4 — Authors’ original file for figure 4 [file 12891_2013_2342_MOESM4_ESM.tif]

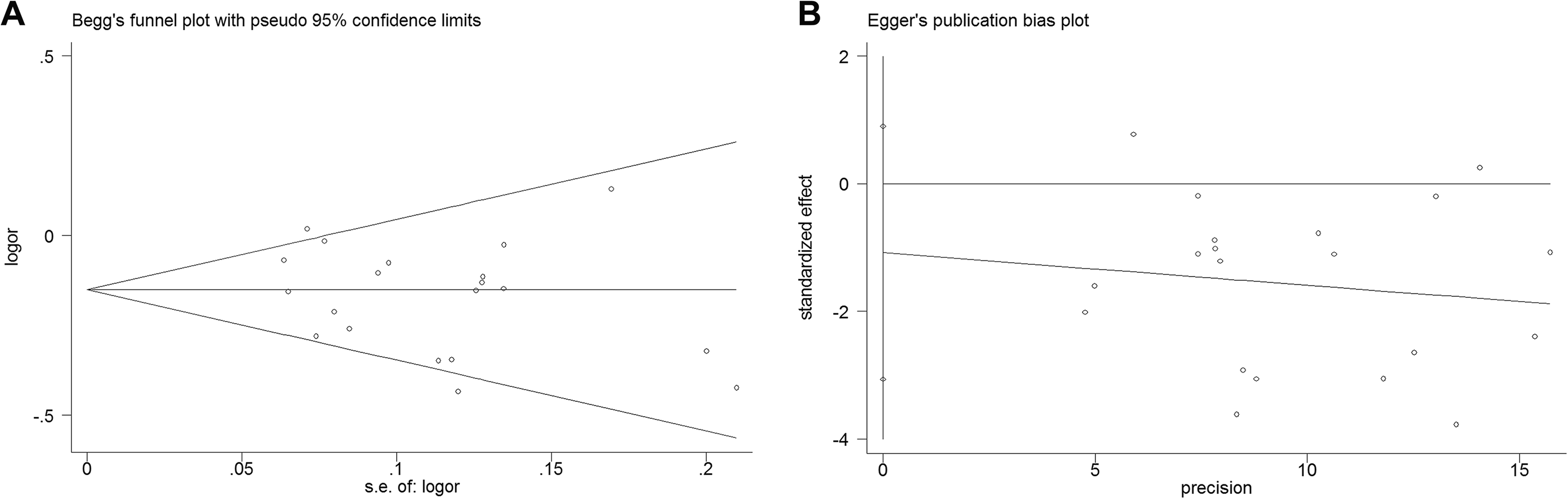

Supplement: Supplementary file 5 — Authors’ original file for figure 5 [file 12891_2013_2342_MOESM5_ESM.tif]
